# Pharmacokinetics of an injectable long-acting formulation of doxycycline hyclate in dogs

**DOI:** 10.1186/1751-0147-54-35

**Published:** 2012-06-08

**Authors:** Lilia Gutiérrez, Zazil-Ha Velasco, Carlos Vázquez, Dinorah Vargas, Héctor Sumano

**Affiliations:** 1 Department of Physiology and Pharmacology, School of Veterinary Medicine, National Autonomous University of Mexico (UNAM), Avenida Universidad 3000, Coyoacán, México City, 04510, Mexico; 2 Center for Research and Advanced Studies in Animal Health, Autonomous University of the State of Mexico (UAEM), Km 14.5 Carretera Toluca-Ixtlahuaca, San Cayetano, Toluca, 50200, Mexico; 3 Department of Physiology and Pharmacology, School of Veterinary Medicine, National Autonomous University of Mexico (UNAM), Avenida Universidad 3000, Coyoacán, Mexico City, 04510, Mexico

**Keywords:** Doxycycline, Dog, Pharmacokinetics, Long-acting, Poloxamer, β-cyclodextrin

## Abstract

Based on its PK/PD ratios, doxycycline hyclate (DOX-h), a time-dependant antibacterial, is ideally expected to achieve both sustained plasma drug concentrations at or slightly above the MIC level for as long as possible between dosing intervals. Pursuing this end, a poloxamer-based matrix was used to produce a long-acting injectable preparation (DOX-h-LA) and its serum concentrations vs. time profile investigated after its SC injection to dogs (≤ 0.3 mL per injection site), and results compared with the oral (PO) and IV pharmacokinetics of DOX-h, prepared as tablet or as freshly made solution. A crossover (4 x 4 x 4) study design was employed with 12 Mongrel dogs, with washout periods of 21 days, and at dose of 10 mg/kg in all cases. DOX-h-LA showed the greatest values for bioavailability (199.48%); maximum serum concentration (Cmax) value was 2.8 ± 0.3 with a time to reach Cmax (Tmax) of 2.11 ± 0.12 h and an elimination half-life of 133.61 ± 6.32 h. Considering minimum effective serum concentration of 0.5 μg/mL, a dose-interval of at least 1 week h can be achieved for DOX-h-LA, and only 48 h and 24 h after the IV or PO administration of DOX-h as a solution or as tablets, respectively. A non-painful small bulge, apparently non-inflammatory could be distinguished at injection sites. These lumps dissipated completely in 30 days in all cases.

## Introduction

Doxycycline (Dox), a semi-synthetic derivative of oxytetracycline, is an inexpensive, potent antibacterial drug commonly used as doxycycline hyclate (DOX-h). It differs from oxytetracycline, chlortetracycline and tetracycline in that it is 5–10 times more lipophilic [1]; hence, it possesses higher tissue penetration, larger volume of distribution and better antimicrobial properties, considering that entry into bacteria is not dependant on an active transportation mechanism as it occurs with the above referred tetracyclines. Additionally, Dox has a more prolonged half-life and a greater plasma protein binding rate [2], both in humans and animals.

The broad spectrum-antimicrobial effects of DOX-h are based on hindering bacterial protein synthesis, by interference with the binding of aminoacyl-tRNA to the mRNA ribosome complex thus arresting growth [3,4]. This mechanism of action has been linked to optimal clinical results when the drug is administered to comply with a time-dependant action [5,6]. That is, serum concentrations of DOX should ideally never be below the MIC at any time during the dosing interval [7]. In dogs, DOX has been listed as drug of choice to treat bacterial infections caused by *Haemobartonella canis, Staphylococus spp, Streptococcus spp, Haemophilus spp, Bordetella bronchiséptica, Mycoplasma spp Leptospira spp, Erclichia canis, Borrelia burgdorferi, Brucella canis, Campylobacter jejuni, Fusobacterium spp*. [1,6,8,9]. To treat diseases causes by these bacteria, DOX is customary administered as tablets once or twice daily in prolongued dosing schemes. However, compliance with long-term dosing is often defective, either by negligence or caused by vomiting and other gastrointestinal adverse drug reactions [2]. Injection of an aqueous preparation of DOX-hyclate is not an option because as it occurs with other tetracyclines, this drug is remarkably tissue-irritating [1,8,10], a fact that also explains the lack of an injectable long-acting (LA) preparation. A possible exception can be found in the poloxamer β-cyclodextrin-based matrix, long acting formulation of doxyclyne (DOX-h-LA) that has been proposed for cows [11] and goats [12]. In these trials both, favorable pharmacokinetics and a minimum irritation at the injection site were found.

Considering the above, the objectives of this study were: establish the pharmacokinetics of the referred experimental preparation of DOX-h-LA after its SC injection at a dose of 10 mg/kg of body weight in volumes of injection not greater than 0.3 mL, and to compare these results with the pharmacokinetics of DOX-h after the oral administration of tablets or the IV injection of a freshly made aqueous solution.

## Material and methods

This study was approved by the Institutional Committee of Research, Care and Use of Experimental Animals (CICUAE), according to de Mexican Official Regulation NOM-062-ZOO-1999. This study was carried out at the Facultad de Medicina Veterinaria y Zootecnia at the Universidad Nacional Autonoma de Mexico, located in Mexico City.

Twelve clinically healthy mongrel dogs 2–9 years old (7 males and 5 bitches), weighing 18 ± 4.2 kg (range: 10–23 kg), were included in this trial. Upon arrival to the Veterinary College, animals were dewormed with an oral ivermectin-praziquantel preparation (Pet-Gard®, from Cpmax Pharmaceuticals, Mexico). Dogs were fed *ad libitum* with commercial pelleted feed (Pro-Plan®, Purina México), and allowed to get accustomed for 15 days to new surroundings in individual-ground 2 x 3 m cages, with free access to clean drinking water and 1 h daily walks. No antibacterial or any other medication was administered for at least 15 days prior to receiving their injection or tablet of DOX-h. To assign dogs to a given treatment a three way crossover model (4 x 4 x 4) was used, with a washout period of 21-days. For all animals, a foster family was ensured after the study.

A subcutaneous LA preparation (100 mg/mL) of doxycycline hyclate was developed (PARFARM Pharmaceuticals, Mexico City, Mexico), under sterile conditions. Inclusion complexes of doxycycline 10% (w/v) with β-cyclodextrin (1:0.1 M) (Cerestar Pharmaceutical Excipients, U.S.A.) were first formed by the kneading method which can be described as follows: β-cyclodextrin (0.1 M) and distilled water were mixed together in a mortar so as to obtain a homogeneous paste. Then, doxycycline (1 M) was added slowly. The mixture was ground for 30 min and an appropriate quantity of water was added to maintain a paste-like consistency. It was then dried in oven at 40-50˚ C for 24 hours. The dried complex was pulverized into a fine powder [13]. The resulting powder was diluted with a solution of 15% propylenglycol - 10% ethyl alcohol in water. Then, this mixture was included in a reverse gel copolymer polyoxypropile–polyoxyethylene (poloxamer) BASF (Mexico City, Mexico) adjusting pH to 7.0 with a phosphate buffer solution under constant stirring at 4° C to reach a final concentration of 10% of doxycycline in 15% poloxamer. The preparation was regarded as ready when a micro-emulsion is formed and this can be pin-pointed when the mixture clarifies (Patent No: 289266, *Instituto Mexicanos de la Propiedad Industrial*; April 25, 2997). Then, 10 ml vials were prepared, stored at 4 °C and utilized during the following week.

Ten percent aqueous solutions were made from powder DOX-h diluted in sterile distilled water immediately before injection. A dose of 10 mg/kg in a volume approximately 1 mL/10 kg of body weight was administered intravenously through the cephalic vein using a catheter No. 20 (Becton, Dickinson and Company, México). The experimental, long-acting preparation of DOX-h developed for this trial (DOX-h-LA), was injected SC at the same dose of 10 mg/kg, but in equally divided volumes never exceeding 0.3 mL per site, in the costal area. Two hundred and fifty mg tablets of doxycycline were administered by concealing them in 200 g pork-sausages and ensuring their complete consumption. Dose was adjusted to 10 mg/kg by scraping the tablet to quadrate to each individual weight. In all three groups adverse drug reactions were sought for with hour to hour observations during the day.

Individual dose vs. pharmaceutical preparation compliance was calculated to have 6.5, 4.2 and 3.8% error from the set dose of 10 mg/kg for tablet, LA and aqueous preparation, respectively, as assessed by determining Dox concentration in all three preparations, taking 4 random test samples of each group.

In order to achieve a close timing interval between administration of the drug and blood sampling from the radial vein, a permanent-heparinized 5-inch long catheter gauge No. 20 (Becton, Dickinson and Company, México City) was placed in each dog and a plastic collar ensured to avoid dogs from reaching the catheter. Three mL blood samples were withdrawn after discarding the first 2 mL of heparinized blood. The times for collection after administration of the drug were: 0, 0.5, 1, 2, 3, 4, 6, 8, 10, 12, 24, 48, 72, 60, 72, 84, 96, 108, 120, and 200 h. Blood samples were immediately centrifuged, plasma recovered, identified, and frozen at -20° C until analyzed within five days.

Serum doxycycline concentrations were determined by the modified agar diffusion analysis, described by Abd El-Aty *et al.[*[14]*]* with *Bacillus cereus* (ATCC-11778) as a test organism grown on Müeller-Hinton agar (MCD LAB, S.A. de C.V., México City). Drug concentrations were determined by comparing the diameter of zones of inhibition with those of various dilutions of the standard prepared in pooled antibacterial-free dog serum by the use of linear regression analysis. The intra-assay coefficient of variance was <4.9 and inter-assay error <4.8. The analytical assay was linear over a range of concentrations from 0.05 to 10 μg/mL,with a percent recovery of 94 ± 2 and a correlation coefficient (r^2^) of 0.97 ± 0.1. Limit of detection was 0.005 μg/mL and limit of quantification was 0.01 μg/mL. A computerized curve-stripping program (PKAnalyst®, Micromath Scientific Software, SLM, USA) was used to fit and analyze the concentration vs. time profiles of each individual dog and mean values later derived. Best fitting models were chosen after analysis with residual sum of squares and the minimal Akaike’s information criterion. For the PO route best fit was obtained using a two compartment models with first–order input and first–order output using (Model 11, r ≥ 0.99), whose formula is:

(1) $$\begin{array}{rl} Concentration\;\left(Time\right)= & \frac{\text{Dose}\cdot {\text{K}}_{\text{AB}}}{Volume\;K_{\mathrm{AB}}-K_{\mathrm{elim}}}e^{-kelim•Time}\\ & -e^{-KAB•Time.} \end{array}$$

For the SC route best fit was obtained using a two compartment models with first–order input and first–order output using (Model 13, r ≥ 0.99), whose formula is:

(2) $$\begin{array}{rl} Concentration(Time)= & Ae^{-\alpha \cdot Time}+Be^{-b\cdot Time}\\ & -{Ce}^{-KAB\cdot Time.} \end{array}$$

The concentration-time curve of DOX-h IV was best fitted using a two compartment model as presented in Model 7 (r ≥ 0.99), with the following formula:

(3) $$Concentration(Time)=Ae^{-\alpha \cdot Time}+Be^{-\beta \cdot Time}$$

Pharmacokinetic variables obtained with PKAnalyst for the IV route were: AUC_0-∞_ = Area under the curve; AUMC_0-∞_ area under the moment curve; RT = retention time; A, B, = zero time intercepts of the distribution and post-distribution phases; α and β = hybrid rate constants for the distribution and elimination phases, respectively; T½β = half-life of the elimination; Cs_0_ = maximum serum concentration at time zero. Other variables, such as Vd_c_ = Volume of the central compartment; Vd_area_ = Volume calculated by the area method; Vdss = apparent volume of distribution at steady-state and CL_B_ = clearance from the body were obtained with standard formulas, as proposed by [15,16].

Pharmacokinetic variables obtained for the extravascular route were: AUC_0-∞_ = Area under the concentration-time curve from zero up to ∞ with extrapolation of the terminal phase; AUMC = Area under the first moment of the concentration-time curve; AUC_t_ = Area under the concentration-time curve calculated by the trapezoidal method; AUMC_t_ = Area under the first moment of the concentration-time curve calculated by the trapezoidal method; RT = Retention time; K½_el_ = Elimination half rate constant from the central compartment; K_½ab_ = Absorption half rate constant from the central compartment; A, B, = zero time intercepts of the distribution and post-distribution phases; α and β = hybrid rate constants for the distribution and elimination phases, respectively; T½β = half-life of the elimination; Cmax = Calculated maximum plasma concentration; T_max_ = Time of maximum plasma concentration; F = Bioavailability.

Two bioavailability (F%) values were calculated using the following equation:

(4) $$F\%=\frac{{\text{AUC}}_{0-\infty }\;e.v.}{AUC_{0-\infty }\;i.v.}X\frac{Dosis\;i.v.}{Dosis\;e.v.}X100$$

Data are presented as mean ± standard deviation of 12 observations for each parameter and for statistical comparisons of Cmax, Tmax, AUC, RT and T½β among groups the Kruskal-Wallis and Dunn-test, were used.

## Results

**Figure 1:**
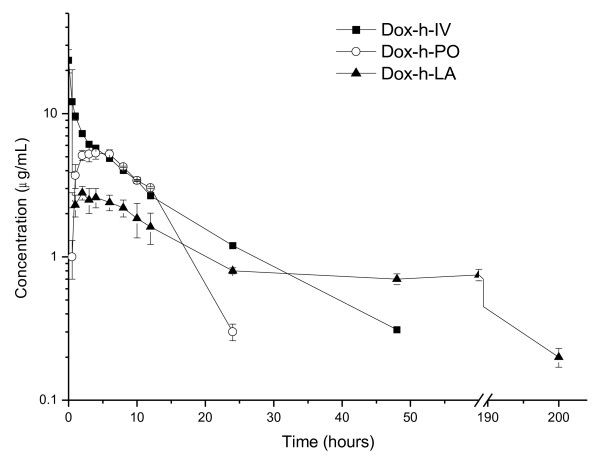
**Mean ± 1 SD plasma concentrations (Log**_**10**_**) vs. time profiles of doxycycline in dogs after a single bolus IV dose of an aqueous preparation of the drug, a single dose of the drug as a tablet and a single SC injection of an experimental long-acting preparation.** Dose was 10 mg/kg in all cases.

Mean ± 1 SE plasma concentrations vs. time profiles of DOX-h in dogs after a single bolus IV dose of the aqueous preparation of the drug, a single dose of the drug as a tablet and after the SC administration of the experimental preparation, are presented in Figure 1. Table 1 summarizes all pharmacokinetic variables obtained, which showed no normal distribution for the three groups. A statistical comparison, made for those variables shared by the three administration routes, is also presented in Table 1.

**Table 1:** Pharmacokinetic variables calculated for doxycycline (10 mg/kg) in dogs through compartmental analysis, after either the IV, PO and SC administration of an aqueous solution, a preparation as tablet or the experimental long acting preparation, respectively

	**Doxycycline IV**	**Doxycycline PO**	**Doxycycline SC**
	**Mean ± SE**	**Mean ± SE**	**Mean ± SE**
AUC_0-∞_ (μg·h/mL)	97.34 ± 7.45^a^	72.89 ± 6.3^b^	194.18 **±** 12.72^c^
AUC_t_ (μg·h/mL)	109.66 ± 8.56^a^	70.33 ± 6.23^b^	129.70 ± 9.56^c^
AUMC_t_ (μg·h^2^/mL)	909.64 ±23.82^a^	820.11 ± 18.63^b^	10453.52 ± 135.78^c^
RT (h)	10.10 ± 1.12^a^	10.22 ± 2.21^a^	166.63 ± 5.58^b^
K½_ab_ (h)	-	1.49 ± 0.07	^-^
K½_el_ (h^-1^)	2.87 ± 0.02^a^	5.59 ± 0.03^b^	45.21 ± 0.74^c^
A (h^-1^)	14.99 ± 0.11^a^	-	2.16 ± 0.03^b^
B (h^-1^)	8.50 ± 0.07^a^	-	0.86 ± 0.01^b^
α (h^-1^)	2.49 ± 0.02^a^	-	0.07 ± 0.004^b^
β (h^-1^)	0.09 ± 0.003^a^	-	0.005 ± 0.001
T½α (h^-1^)	0.28 ± 0.01	-	9.30 ± 0.36 ^b^
T½β (h^-1^)	7.44 ± 0.06 ^a^	-	133.61 ± 6.32 ^b^
Vd_c_ (L/kg)	6.37 ± 0.76	-	^-^
Vd_area_ (L/kg)	1.49 ± 0.27	-	^-^
Vd_ss_ (L/kg)	11.34 ± 1.24	-	^-^
Cs_0_ (μg/mL)	23.54 ± 4.32	-	-
Cmax (μg/mL)	-	5.58 ± 0.5^a^	2.8 ± 0.3^b^
T_max_ (h)	-	3.88 ± 0.4^a^	2.11 ± 0.12^b^
Cl_B_ (mL/kg · h^-1^)	134 ± 8	-	-
F (%)^*^	-	74.88	199.48

^a,b,c,d^The values within a row with no common superscript differ significantly (P < 0.05).

AUC_0-∞_ = area under the concentration-time curve from zero up to ∞ with extrapolation of the terminal phase; AUMC = area under the first moment of the concentration-time curve; AUC_t_ = area under the concentration-time curve calculated by the trapezoidal method; AUMC_t_ = area under the first moment of the concentration-time curve calculated by the trapezoidal method; RT = retention time; K½_el_ = elimination half rate constant from the central compartment; K_½ab_ = absorption half rate constant from the central compartment; A, B, = zero time intercepts of the distribution and post-distribution phases; α and β = hybrid rate constants for the distribution and elimination phases, respectively; T½β = half-life of the elimination; Vd_c_= volume of the central compartment; Vd_area_= Volume calculated by the area method; Vdss = apparent volume of distribution at steady-state; Cs_0_ = calculated maximum plasma concentration at zero time; Cmax = calculated maximum plasma concentration; T_max_ = time of maximum plasma concentration; Cl_B_ = clearance from the body; F = bioavailability.

Comparisons for AUC, RT and T½β, showed that all these variables were statistically larger in group DOX-h-LA as compared to groups DOX-h-IV or DOX-h-PO (P ranging from <0.05 to <0.001). Bioavailability values for the DOX-h-LA group were 199.48%, while the same variable for the DOX-h-PO group was 74.88%. The half rate constant from the central compartment obtained were 45.21 ± 0.74 h for DOX-h-LA 5.59 ± 0.03 h for the DOX-h-PO, and 2.87 ± 0.02 h for DOX-h-IV. Difference among these statistical means half rate constant from the central compartment obtained for Dox-h-LA was statistically longer (P < 0.001).

Based on an arbitrary minimal therapeutic concentration a comparative assessment was made of the length of time for which a given serum concentration of doxyxycline was maintained. Chosen value was 0.12 μg/mL [1,17,18]. Under this setting, DOX-h-IV maintained suitable serum concentrations for 8 h; DOX-h-PO for 22 h and DOX-h-LA extended these time-periods to approximately 200 h. The time at which serum concentrations of Dox were at an appropriate level were statistically different when comparing DOX-h-LA with either DOX-h-IV and DOX-h-PO (P < 0.001).

No tissue samples were obtained from injection sites; yet, no typical inflammatory response was observed at the injection site with the DOX-h-LA preparation. However, a well defined apparently painless bulge remained detectable for up to 30 days. These lumps began with an approximate diameter size of 1.5 - 2 cm and disappeared steadily. These bulges are not believed to be inflammatory responses, but rather space occupied lumps caused by the poloxamer when becoming gel at body temperature. After 30 days no lesion could be detected. Animals did not show any unusual sign of pain or discomfort with the long-acting preparation either when injected or afterwards.

## Discussion

The quantitative/qualitative microbiological agar diffusion technique used in this trial to determine serum concentrations of doxycycline has been regarded as sufficiently reliable to replace analytical conclusions derived from high performance liquid chromatography [19]. Furthermore, because it determines the active fraction(s) of the drug, it offers more clinically meaningful data than concentration values derived from purely chemical methods. In turn, this allows straight forward speculations on the relationships between serum concentrations, clinical efficacy and dosing intervals for specific pathogens.

The pharmacokinetics of DOX-h has been reported in dogs following its IV administration [20]. Variables described by these authors and reported values in this study are very much in agreement. For example Cp_0_ with a 10 mg/kg dose was 23.54 ± 4.32 in this trial, and 11.77 ± 2.26 μg/mL in the referred study with a dose of 5  mg/kg;  AUC  was  109.66 ± 8.56  μg.h/mL  and  52.76 ± 15.34  μg.h/mL for obtained and reported results, respectively and elimination half-life was 0.09 h in both cases. The half-life value obtained for DOX-h-IV in this study falls within the range documented for other species varying from 4.2 h to 16.6 h [10,21-24]. However, no studies are available on the pharmacokinetics of neither the oral administration of the drug as tablets, nor the SC injection of a long-acting preparation, in dogs.

The preparation DOX-h-PO resulted in *C*_max_ values twice that of the DOX-h-LA group (5.58 ± 0.5 μg/mL vs. 2.8 ± 0.3 μg/mL), and T_max_ occurred in 3.9 ± 0.4 and 2.11 ± 0.1 h, respectively. After Cmax, serum concentrations of DOX declined slowly with a K½_el_ of 45.21 h in the DOX-h-LA group, which is a value considerably larger than the corresponding one in PO group (K½_el_ of 5.59 h). Predictably for a highly lipid soluble drug, a high apparent volume of distribution area was achieved after IV administration of the drug (Vd_area_ = 1.49 ± 0.27 L/kg. Retention time (RT) was considerably larger in the DOX-h-LA group (166.63 ±5.58 h) vs. 10.10 ± 1.12 h and 10.22 ± 2.21 h for the IV and PO groups, respectively.

Absolute bioavailability of doxycycline for the DOX-h-LA group was 199.48%. Bioavailabilities as high as the one reported in this manuscript for Dox-h-LA are not uncommon for formulations with prolonged absorption, exhibiting flip-flop kinetics [17]. It has been proposed that this phenomenon is either a fact or a flaw in sampling or handling of samples, experimental design misconceptions, analytical deficiencies or miscalculations [15]. For example, it can be that depletion of doxycycline after 24 h is based on fewer data points and bioavailability is artificially overestimated. Yet, a careful review of these and other factors listed by Toutain and Bousquet-Mélou [15] offered no clear explanation as to the large value of F obtained by the DOX-h-LA preparation injected SC in this study, excepting, a recycling phenomenon due to the noticeably high lipid solubility of this drug [25,26], and its controlled release from the experimental preparation.

A flip – flop pharmacokinetic model can be established by the overall appearance of the serum concentration vs. time profile of the drug, considering that the rate of absorption is slower than the rate of elimination; this is not always a clear-cut panorama. According to [27] a flip-flop model can be recognized when the plasma concentration-time profile tends either to closely parallel rate of absorption. This provides a simple and effective way to visualize the shape of the rate of absorption profile. Thus applying the following equation, a "flip-flop" condition may be demonstrated for this preparation:

(5) RateofAbsorption=Vz(KC+(ΔC/Δt))

Where, Vz is the terminal exponential volume of distribution, K is the terminal disposition rate constant once drug absorption is complete (best determined from i.v. dosing), C is the plasma concentration at time t and ΔC is the change in plasma concentration over the time interval Δt. For DOX-h-LA plasma concentration-time data at 48 and 72 hours, ΔC/Δt = 0.033 μg/mL/hour. At the midpoint of this time period (60 h), (K)(C) = 2.1 μg/mL/hr. Since KC > ΔC/Δt*,* rate of absorption ≈ rate of elimination, a "flip-flop" condition exists and the DOX-h-LA here described can be regarded as a true long-acting one.

The poly(ethylene oxide)–poly(propylene oxide)–poly(ethylene oxide) block copolymer (poloxamer) was used as delivery vehicle-matrix, because it improves solubility, reduces hydrolytic degradation, achieves controlled release and often results in improved bioavailability [28,29]. In part these effects are obtained by its reversed temperature-dependant gelation. That is, the poloxamer-based formulation exhibits low viscosity at room temperature and becomes gel at body temperature [Rehman *et al.*. It is here postulated that DOX-h/β-cyclodextrin complexes may diminish or prevent tissue irritation by reducing the local concentration of free drug below the irritancy threshold [ [30]; 30], while enhancing absorption rate with a priming phase [30,31]. The benefits of controlled delivery of drugs include: the maintenance of serum drug concentration at an optimal therapeutic level for a prolonged time-interval, reduction in handling and consequently, a possible improvement in drug-administration compliance [32]. In this context, the DOX-h-LA preparation here described was capable of providing, with a single SC injection, useful serum concentrations of this antibacterial drug for 5 to 7 days, and no side effects were observed. Nevertheless, a complete toxicological analysis is necessary if this drug preparation is to be used in dogs. Perhaps a key issue to study further would be local tissue reactions that may occur in skin sensitive dogs after repeated administrations of this experimental preparation.

Although, it has been stated that high concentrations of doxycycline *in vitro*, equivalent to 8 to 16 times the value of an average MIC, could turn doxycycline into a concentration-dependant antibacterial [33] cardiac toxicity of this drug has been reported in calves with a single overdose 5-10 times larger with plasma concentrations ranging from 50 - 100 μg/ml. Hence, seeking large Cmax values appears to be an unsafe approach [34,35] and DOX should be considered a time-dependant antibacterial drug [33]. In that context PK/PD compliance can be achieved when serum concentrations of the drug are barely above or at the minimal inhibitory concentration (MIC) level of the involved pathogen, but for as long as possible within the dosing interval [36]. Values of MIC that can be adopted in this trial can be set from 0.5 to 1.5 μg/mL [36]. Hence, the length of time in which minimum therapeutic concentrations can be achieved with DOX-h-PO varies from 12 to 24 h, and for DOX-h-LA this interval is extended to at least 7 days. Also, a PK/PD index accepted as predictor of therapeutic efficacy for tetracyclines as a group in humans is the ratio of AUC at steady state (AUCss)/MIC [37]. AUCss was not evaluated in this study. However if a pathogen is susceptible at 1.5 μg/mL, AUC DOX-h-LA/MIC ratio is 86.04 for DOX-h-LA, and only 46.72 for AUC_DOX-h-PO_/MIC. Considering the above and the fact that serum concentrations of doxycycline are ideally compelled to be never below the MIC at any time during the dosing interval [38], it is safe to regard DOX-h-LA as a drug preparation that possesses better PK/PD ratios to control bacterial diseases in dogs as compared to tablets or an IV solution. In these latter cases serum concentrations of the drug will show daily peaks and troughs as opposed to a more steady serum concentrations achieved with DOX-h-LA.

Summarizing, DOX-h-LA is a preparation that optimizes the use of doxycycline in dogs in terms of PK/PD ratio congruency, and it is likely that it may also improve prescription compliance. Nevertheless, clinical trials and toxicological studies are needed to assess if this preparation can be regarded as potentially useful in this species.

## Competing interests

The authors declare that they have no competing interests.

## Authors’ contribution

All authors read and approved the final manuscript.
